# Exogenous Proline and Glycine Betaine Mediated Upregulation of Antioxidant Defense and Glyoxalase Systems Provides Better Protection against Salt-Induced Oxidative Stress in Two Rice (*Oryza sativa* L.) Varieties

**DOI:** 10.1155/2014/757219

**Published:** 2014-06-03

**Authors:** Mirza Hasanuzzaman, Md. Mahabub Alam, Anisur Rahman, Md. Hasanuzzaman, Kamrun Nahar, Masayuki Fujita

**Affiliations:** ^1^Department of Agronomy, Faculty of Agriculture, Sher-e-Bangla Agricultural University, Sher-e-Bangla Nagar, Dhaka 1207, Bangladesh; ^2^Laboratory of Plant Stress Responses, Department of Applied Biological Science, Faculty of Agriculture, Kagawa University, 2393 Ikenobe, Miki-cho, Kita-gun, Kagawa 761-0795, Japan; ^3^Department of Agricultural Botany, Faculty of Agriculture, Sher-e-Bangla Agricultural University, Sher-e-Bangla Nagar, Dhaka 1207, Bangladesh

## Abstract

The present study investigates the roles of exogenous proline (Pro, 5 mM) and glycine betaine (GB, 5 mM) in improving salt stress tolerance in salt sensitive (BRRI dhan49) and salt tolerant (BRRI dhan54) rice (*Oryza sativa* L.) varieties. Salt stresses (150 and 300 mM NaCl for 48 h) significantly reduced leaf relative water (RWC) and chlorophyll (chl) content and increased endogenous Pro and increased lipid peroxidation and H_2_O_2_ levels. Ascorbate (AsA), glutathione (GSH) and GSH/GSSG, ascorbate peroxidae (APX), monodehydroascorbate reductase (MDHAR), dehydroascorbate reductase (DHAR), glutathione reductase (GR), glutathione peroxidase (GPX), catalase (CAT), and glyoxalase I (Gly I) activities were reduced in sensitive variety and these were increased in tolerant variety due to salt stress. The glyoxalase II (Gly II), glutathione S-transferase (GST), and superoxide dismutase (SOD) activities were increased in both cultivars by salt stress. Exogenous Pro and GB application with salt stress improved physiological parameters and reduced oxidative damage in both cultivars where BRRI dhan54 showed better tolerance. The result suggests that exogenous application of Pro and GB increased rice seedlings' tolerance to salt-induced oxidative damage by upregulating their antioxidant defense system where these protectants rendered better performance to BRRI dhan54 and Pro can be considered as better protectant than GB.

## 1. Introduction

Crop plants, as sessile organisms, face a number of environmental adversities termed as abiotic stress which includes soil salinity, water deficit, extremely high or low temperatures, toxic metals, waterlogging, elevated ozone, and ultraviolet radiation, which all create a barrier for proper growth, metabolism, and productivity of crop plants [1–10]. Among the environmental stresses soil salinity is a widespread environmental problem that has been found to affect more than 77 million hectares or 5% of the cultivable land of the universe [11, 12]. Salinity adversely affects the plant growth and productivity. The yield reduction due to salt stress may account for substantial reduction of the average yield of major crops by more than 50% [13]. The nature of damages due to salt stress is very complex because it causes both osmotic stress and ionic toxicity [7]. At molecular level, one of the common events in plants grown under salt stress is the considerable increases in reactive oxygen species (ROS) such as singlet oxygen (^1^O_2_), superoxide radical (O_2_^•−^), hydrogen peroxide (H_2_O_2_), and hydroxyl radical (^•^OH) [2]. However, the production of ROS greatly depends on the degree and duration of the imposition of stress and types of crop as well [2]. Considering the destructive effects of salt-induced oxidative stress in plants it is crucial to keep the ROS level below the toxic limit. Plants always try to keep well-developed enzymatic and nonenzymatic antioxidant defense system ready to encounter the deleterious effects of ROS [2]. The enzymatic system includes the four enzymes of the ascorbate-glutathione (AsA-GSH) cycle: ascorbate peroxidase (APX), monodehydroascorbate reductase (MDHAR), dehydroascorbate reductase (DHAR), and glutathione reductase (GR) as well as other enzymes like superoxide dismutase (SOD), catalase (CAT), glutathione peroxidase (GPX), and glutathione* S*-transferase (GST). The nonenzymatic antioxidants include ascorbic acid (AsA), glutathione (GSH), phenolic compounds, alkaloids, nonprotein amino acids, and *α*-tocopherols. They act together in scavenging or detoxifying ROS and subsequent protection of plant cells from oxidative damage [2, 4]. However, this system acts differently in different plant species and cultivars and it was observed that the enhancement of the antioxidant defense system is often correlated with salt stress tolerance [4, 7]. Methylglyoxal (MG) is another highly reactive cytotoxic which is produced largely under abiotic stress including salinity [14, 15] and leads to damages to proteins, lipids, and DNA [16, 17]. In line with antioxidant defense system plants also possess glyoxalase system consisting of two enzymes: glyoxalase I (Gly I) and glyoxalase II (Gly II); those can detoxify MG. Enzymes of the glyoxalase system are found to regulate environmental stresses including salinity as reported in many plant studies [2, 3, 10, 18]. It was reported that the coordinated upregulation of both the antioxidant defense and glyoxalase systems is necessary to attain significant tolerance to oxidative stress [2, 6].

It is an urgent task of plant biologists to explore suitable mechanisms of developing salt tolerant crop plants that can produce sufficient yield under adverse condition. In recent decades, many researchers have been trying to find the ways to alleviate salt stress or to overcome salt injury in plants. Among them exogenous application of substances such as osmoprotectants, phytohormones, antioxidants, and trace elements came to attention in recent times [4, 19, 20].

In response to various environmental stresses, plants demonstrate a variety of adaptive mechanisms to counteract them. Since one of the primary responses under salt stress is osmotic adjustment, compatible solutes such as proline (Pro) and glycine betaine (GB) are very common to be accumulated during salt stress and play a fundamental role in osmotic adjustment in plants [21]. These compatible solutes are accumulated in the cytosol without disturbing intracellular biochemistry, which ameliorate the detrimental effects of salinity [22–25].

In many plant species increased accumulation of Pro and GB were observed as an indicator of salt stress tolerance [22, 26]. However, most of the plants, especially under elevated levels of salt, cannot synthesize sufficient amount of these osmoregulators. In many recent reports exogenous applications of Pro and GB were found to act as protectants under salt stress [12, 25, 27]. Besides osmoprotection, Pro and GB also showed their roles in elimination of oxidative stress by triggering the antioxidant defense and also glyoxalase system [25, 28–32].

Although there are several reports on the role of Pro and GB in salt stress tolerance in terms of growth and physiology, few investigations have been done on the effects of exogenous Pro and GB on both antioxidant defense and the glyoxalase system in rice. Therefore, we investigated the protective effects of these osmoprotectants on the antioxidant defense and glyoxalase systems in rice seedlings grown under saline media. We also investigated the comparative performance of two modern rice varieties differing in their salt tolerance to know their actual adaptive mechanisms under salt stress with or without osmoprotectants.

## 2. Materials and Methods

### 2.1. Plant Materials and Stress Treatments

Seeds of two rice (*Oryza sativa* L.) cultivars, cv. BRRI dhan49 (salt sensitive) and cv. BRRI dhan54 (salt tolerant) were collected from Bangladesh Rice Research Institute (BRRI) and surface-sterilized with 70% ethanol for 10 min followed by washing several times with sterilized distilled water. Seeds were then soaked for 24 h in the dark. Seeds were sown on plastic nets upon plastic beakers containing distilled water and kept in the dark at 28 ± 2°C for germination. After 48 h, uniformly germinated seeds were transferred to growth chamber with controlled conditions (light intensity, 100 *μ*mol m^−2^ s^−1^; temperature, 25 ± 2°C; relative humidity, 65–70%) and during the growing period hyponex solution (Hyponex, Japan) was used as nutrient. Two levels of salt stresses (150 and 300 mM NaCl) were imposed on fourteen-day-old rice seedlings with, without 5 mM proline [l (-) Proline, Wako, Japan] and betaine (Betaine, Wako, Japan), and where these protectants were sprayed twice a day mixing with the wetting agent 0.02% Tween 20 (Tween 20, Wako, Japan). Control plants were grown with Hyponex solution only. Data were taken after 48 h of NaCl treatment. The experiment was repeated three times (*n* = 3) under the same conditions.

### 2.2. Measurement of Relative Water Content

Relative water content (RWC) was measured according to Barrs and Weatherley [33]. Leaf laminas were weighed (fresh wt, FW) and then immediately floated on distilled water in a petri dish for 8 h in the dark. Turgid weights (TW) were obtained after drying excess surface water with paper towels. Dry weights (DW) were measured after drying at 80°C for 48 h. The calculation was done using the following formula:

(1) $$\begin{array}{c} \text{RWC}\;\left(\%\right)=\frac{\text{FW}-\text{DW}}{\text{TW}-\text{DW}}\times 100. \end{array}$$

### 2.3. Determination of Chlorophyll Content

Chlorophyll content was determined by homogenizing leaf samples (0.5 g) with 10 mL of acetone (80% v/v) followed by centrifuging at 5,000 ×g for 10 min. The absorbance was measured with a UV-visible spectrophotometer at specified wave length and chl contents were calculated using the equations proposed by Arnon [34].

### 2.4. Determination of Proline Content

Free proline in leaf tissues was appraised following the protocol of Bates et al. [35]. Fresh leaf tissue (0.5 g) was homogenized in 10 mL of 3% sulfosalicylic acid in ice. The homogenate was centrifuged at 11,500 ×g for 15 min. Two mL of the filtrate was mixed with 2 mL of acid ninhydrin and 2 mL of glacial acetic acid. After incubation at 100°C for 1 h it was cooled and 4 mL of toluene was added. The optical density of the chromophore containing toluene was read spectrophotometrically at 520 nm using toluene as a blank. The amount of Pro was determined by comparison with a standard curve.

### 2.5. Measurement of Lipid Peroxidation

The level of lipid peroxidation was measured by estimating MDA, using thiobarbituric acid (TBA) as the reactive material following the method of Heath and Packer [36] with slight modifications. The leaf samples (0.5 g) were homogenized in 3 mL 5% (w/v) trichloroacetic acid (TCA) and the homogenate was centrifuged at 11,500 ×g for 10 min. One mL supernatant was mixed with 4 mL of TBA reagent (0.5% of TBA in 20% TCA). The reaction mixture was heated at 95°C for 30 min in a water bath and then quickly cooled in an ice bath and centrifuged at 11,500 ×g for 15 min. The absorbance of the colored supernatant was measured at 532 nm and was corrected for nonspecific absorbance at 600 nm. The concentration of MDA was calculated by using the extinction coefficient of 155 mM^−1^cm^−1^ and expressed as nmol of MDA g^−1^ fresh weight.

### 2.6. Measurement of H_2_O_2_

H_2_O_2_ was assayed according to the method described by Yu et al. [37]. H_2_O_2_ was extracted by homogenizing 0.5 g of leaf samples with 3 mL of 50 mM potassium-phosphate (K-P) buffer (pH 6.5) at 4°C. The homogenate was centrifuged at 11,500 ×g for 15 min. Three milliliter of supernatant was mixed with 1 mL of 0.1% TiCl_4_ in 20% H_2_SO_4_ (v/v) and kept in room temperature for 10 min. After that the mixture was again centrifuged at 11,500 ×g for 15 min. The optical absorption of the supernatant was measured spectrophotometrically at 410 nm to determine the H_2_O_2_ content (*Є* = 0.28 *μ*M^−1^cm^−1^) and expressed as nmol g^−1^ fresh weight.

### 2.7. Extraction and Measurement of Ascorbate and Glutathione

Rice leaves (0.5 g fresh weight) were homogenized in 3 mL ice-cold acidic extraction buffer (5% meta-phosphoric acid containing 1 mM EDTA) using a mortar and pestle. Homogenates were centrifuged at 11,500 ×g for 15 min at 4°C and the supernatant was collected for analysis of ascorbate and glutathione.

Ascorbate content was determined following the method of Huang et al. [38] with some modifications. The supernatant was neutralized with 0.5 M K-P buffer (pH 7.0). The AsA was assayed spectrophotometrically at 265 nm in 100 mM K-P buffer (pH 7.0) with 0.5 unit of ascorbate oxidase (AO). A specific standard curve with AsA was used for quantification.

The glutathione pool was assayed according to previously described methods [37] with modifications [39] utilizing 200 *μ*L of aliquots of supernatant neutralized with 300 *μ*L of 0.5 M K-P buffer (pH 7.0). Based on enzymatic recycling, GSH is oxidized by 5,5′-dithio-bis (2-nitrobenzoic acid) (DTNB) and reduced by NADPH in the presence of GR, and glutathione content is evaluated by the rate of absorption changes at 412 nm of 2-nitro-5-thiobenzoic acid (NTB) generated from the reduction of DTNB. GSSG was determined after removal of GSH by 2-vinylpyridine derivatization. Standard curves with known concentrations of GSH and GSSG were used. The content of GSH was calculated by subtracting GSSG from total GSH.

### 2.8. Determination of Protein

The protein concentration of each sample was determined following the method of Bradford [40] using BSA as a protein standard.

### 2.9. Enzyme Extraction and Assays

Using a pre-cooled mortar and pestle, 0.5 g of leaf tissue was homogenized in 1 mL of 50 mM ice-cold K-P buffer (pH 7.0) containing 100 mM KCl, 1 mM ascorbate, 5 mM *β*-mercaptoethanol and 10% (w/v) glycerol. The homogenates were centrifuged at 11,500 ×g for 10 min and the supernatants were used for determination of enzyme activity. All procedures were performed at 0–4°C.

LOX (EC 1.13.11.12) activity was estimated according to the method of Doderer et al. [41] by monitoring the increase in absorbance at 234 nm using linoleic acid as a substrate. The activity was calculated using the extinction coefficient (25 mM^−1^ cm^−1^) and expressed as units (1 nmol of substrate oxidized per minute) per mg protein.

SOD (EC 1.15.1.1) activity was estimated according to Beyer and Fridovich [42] which was based on xanthine-xanthine oxidase system. The reaction mixture contained K-P buffer (50 mM), NBT (2.24 mM), catalase (0.1 units), xanthine oxidase (0.1 units), xanthine (2.36 mM), and enzyme extract. Catalase was added to avoid the H_2_O_2_-mediated possible inactivation of CuZn-SOD. SOD activity was expressed as units (amount of enzyme required to inhibit NBT reduction by 50%) min^−1^ mg^−1^ protein.

CAT (EC: 1.11.1.6) activity was measured according to the method of Hasanuzzaman et al. [2] by monitoring the decrease of absorbance at 240 nm for 1 min caused by the decomposition of H_2_O_2_. The reaction mixture contained 50 mM K-P buffer (pH 7.0), 15 mM H_2_O_2_ and enzyme solution in a final volume of 700 *μ*L. The activity was calculated using the extinction coefficient of 39.4 M^−1^cm^−1^.

APX (EC: 1.11.1.11) activity was assayed following the method of Nakano and Asada [43]. The reaction buffer solution contained 50 mM K-P buffer (pH 7.0), 0.5 mM AsA, 0.1 mM H_2_O_2_, 0.1 mM EDTA, and enzyme extract in a final volume of 700 *μ*L. The activity was measured by observing the decrease in absorbance at 290 nm for 1 min using an extinction coefficient of 2.8 mM^−1^cm^−1^.

MDHAR (EC: 1.6.5.4) activity was determined by the method of Hossain et al. [44]. The reaction mixture contained 50 mM Tris-HCl buffer (pH 7.5), 0.2 mM NADPH, 2.5 mM AsA, 0.5 unit of AO and enzyme solution in a final volume of 700 *μ*L. The activity was calculated from the change in ascorbate at 340 nm for 1 min using an extinction coefficient of 6.2 mM^−1^cm^−1^.

DHAR (EC: 1.8.5.1) activity was determined by the procedure of Nakano and Asada [43]. The reaction buffer contained 50 mM K-P buffer (pH 7.0), 2.5 mM GSH, and 0.1 mM DHA. The activity was calculated from the change in absorbance at 265 nm for 1 min using extinction coefficient of 14 mM^−1^cm^−1^.

GR (EC: 1.6.4.2) activity was measured by the method of Hasanuzzaman et al. [3]. The reaction mixture contained 0.1 M K-P buffer (pH 7.0), 1 mM EDTA, 1 mM GSSG, 0.2 mM NADPH, and enzyme solution in a final volume of 1 mL. The decrease in absorbance at 340 nm was recorded for 1 min. The activity was calculated using an extinction coefficient of 6.2 mM^−1^cm^−1^.

GST (EC: 2.5.1.18) activity was determined spectrophotometrically by the method of Hossain et al. [45] with some modifications. The reaction mixture contained 100 mM Tris-HCl buffer (pH 6.5), 1.5 mM GSH, 1 mM 1-chloro-2,4-dinitrobenzene (CDNB), and enzyme solution in a final volume of 700 *μ*L. The increase in absorbance was measured at 340 nm for 1 min. The activity was calculated using the extinction coefficient of 9.6 mM^−1^cm^−1^.

GPX (EC: 1.11.1.9) activity was measured as described by Elia et al. [46] with slight modification using H_2_O_2_ as a substrate. The reaction mixture consisted of 100 mM K-P buffer (pH 7.0), 1 mM EDTA, 1 mM NaN_3_, 0.12 mM NADPH, 2 mM GSH, 1 unit GR, 0.6 mM H_2_O_2_, and 20 *μ*L of sample solution. The oxidation of NADPH was recorded at 340 nm for 1 min and the activity was calculated using the extinction coefficient of 6.62 mM^−1^cm^−1^.

Glyoxalase I (EC: 4.4.1.5) assay was carried out according to Hasanuzzaman et al. [2]. Briefly, the assay mixture contained 100 mM K-P buffer (pH 7.0), 15 mM magnesium sulfate, 1.7 mM GSH and 3.5 mM MG in a final volume of 700 *μ*L. The increase in absorbance was recorded at 240 nm for 1 min. The activity was calculated using the extinction coefficient of 3.37 mM^−1^cm^−1^.

Glyoxalase II (EC: 3.1.2.6) activity was determined according to the method of Principato et al. [47] by monitoring the formation of GSH at 412 nm for 1 min. The reaction mixture contained 100 mM Tris-HCl buffer (pH 7.2), 0.2 mM DTNB and 1 mM* S*-d-lactoylglutathione (SLG) in a final volume of 1 mL. The activity was calculated using the extinction coefficient of 13.6 mM^−1^cm^−1^.

### 2.10. Statistical Analysis

All data obtained were subjected to analysis of variance (ANOVA) and the mean differences were compared by a Duncan's multiple range test (DMRT) using XLSTAT v.2013.5.03 software [48]. Differences at *P* < 0.05 were considered significant.

## 3. Results

**Figure 1:**
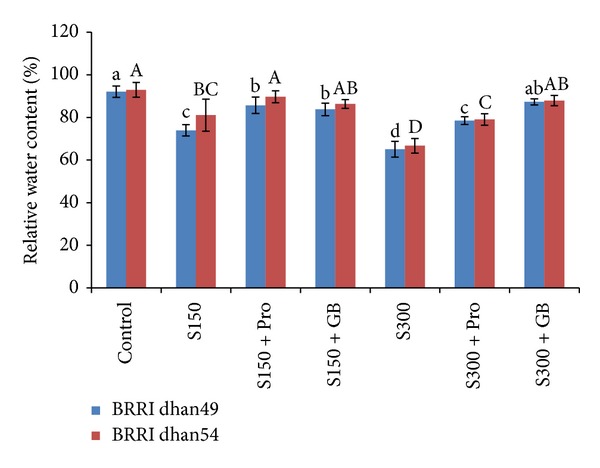
Leaf relative water content in salt sensitive and salt tolerant rice seedlings induced by exogenous proline and glycine betaine under salt stress. S_150_ and S_300_ indicate 150 mM NaCl and 300 mM NaCl, respectively. Pro and GB indicate 5 mM proline and glycine betaine spray, respectively. Mean (±SD) was calculated from three replicates for each treatment. Bars with different letters (small letters for BRRI dhan49 and capital letters for BRRI dhan54) are significantly different at *P* ≤ 0.05 applying DMRT.

Upon exposure to salt, stress leaf RWC decreased significantly in both rice varieties when compared to their controls (Figure 1). However, decline in RWC was lower in salt tolerant cultivar BRRI dhan54 as compared to salt sensitive BRRI dhan49. At 150 mM of NaCl it was decreased by 19 and 12% in BRRI dhan49 and BRRI dhan54, respectively over control, while at 300 mM NaCl the RWC decreased by 29 and 28% (Figure 1). The application of Pro and GB effectively maintained the RWC in salt stressed seedlings. In BRRI dhan49, Pro could increase RWC by 16 and 21% in seedlings exposed to 150 and 300 mM NaCl, respectively, while GB could increase the RWC by 13 and 34%. In case of BRRI dhan54, the increases were 10 and 20% at 150 mM NaCl and 6 and 34% at 300 mM NaCl (Figure 1).

**Figure 2:**
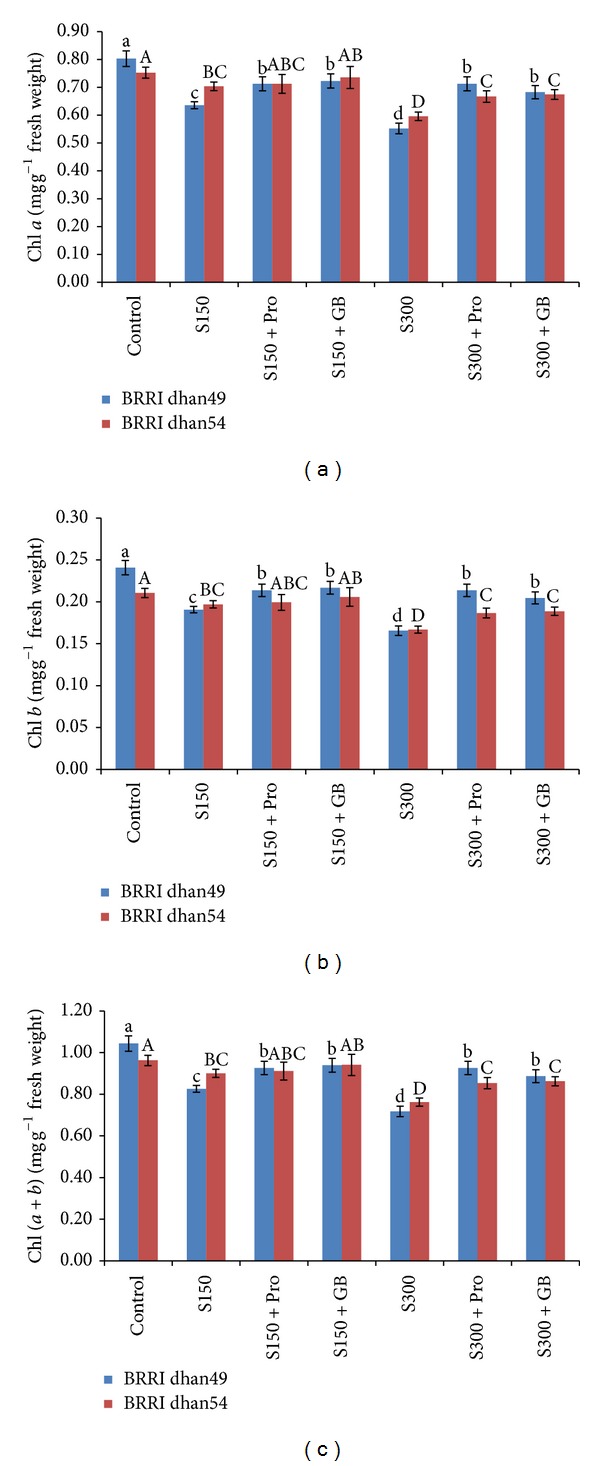
(a) chl *a*, (b) chl *b*, and (c) chl (*a* + *b*) content in salt sensitive and salt tolerant rice seedlings induced by exogenous proline and glycine betaine under salt stress. S_150_ and S_300_ indicate 150 mM NaCl and 300 mM NaCl, respectively. Pro and GB indicate 5 mM proline and glycine betaine spray, respectively. Mean (±SD) was calculated from three replicates for each treatment. Bars with different letters (small letters for BRRI dhan49 and capital letters for BRRI dhan54) are significantly different at *P* ≤ 0.05 applying DMRT.

Leaf chl contents were decreased markedly upon exposure to salt stress. In salt sensitive BRRI dhan49, chl *a* content decreased by 21 and 31% at 150 and 300 mM NaCl while in salt tolerant BRRI dhan54 chl *a* content decreased by 6 and 20% only (Figure 2(a)). On the other hand, when the seedlings were supplemented with exogenous Pro and GB chl *a* content significantly increased in BRRI dhan49 at any level of salt; however, in BRRI dhan54 the increment was observed at 300 mM NaCl only (Figure 2(a)). In BRRI dhan49, chl *b* content was decreased by 21 and 31% at 150 and 300 mM NaCl, while in BRRI dhan54 it was decreased by 6 and 21%, respectively (Figure 2(b)). Exogenous Pro and GB could maintain chl* b* content higher compared to the seedlings grown under salt stress. However, the increment was higher in salt sensitive BRRI dhan49 than salt tolerant BRRI dhan54 (Figure 2(b)). In both rice cultivars chl (*a* + *b*) content markedly decreased upon exposure to salt stress. In BRRI dhan49 chl (*a* + *b*) content decreased by 20 and 31% compared to control while in BRRI dhan54 it decreased by 6 and 21% (Figure 2(c)). In salt sensitive BRRI dhan49 exogenous Pro and GB increased the chl (*a* + *b*) content in the seedlings exposed to any levels of salt, while in salt tolerant the increase was observed in the seedlings exposed to 300 mM NaCl only (Figure 2(c)).

**Figure 3:**
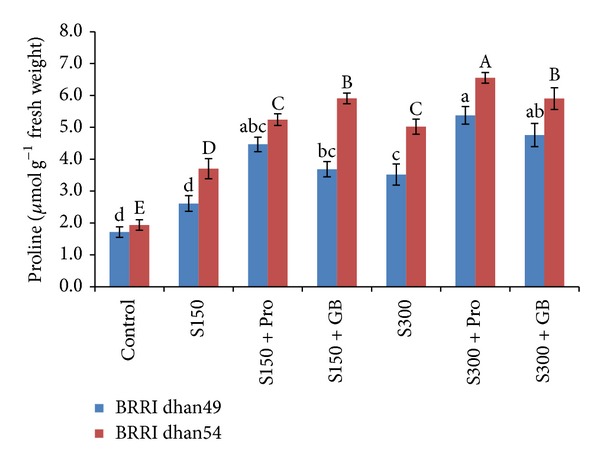
Proline content in salt sensitive and salt tolerant rice seedlings induced by exogenous proline and glycine betaine under salt stress. S_150_ and S_300_ indicate 150 mM NaCl and 300 mM NaCl, respectively. Pro and GB indicate 5 mM proline and glycine betaine spray, respectively. Mean (±SD) was calculated from three replicates for each treatment. Bars with different letters (small letters for BRRI dhan49 and capital letters for BRRI dhan54) are significantly different at *P* ≤ 0.05 applying DMRT.

Salt stress caused a marked increase in endogenous Pro content in rice seedlings of any variety; however, the increment was higher at salt tolerant BRRI dhan54 compared to salt sensitive BRRI dhan49. In BRRI dhan49 Pro content increased by 52 and 105% at 150 and 300 mM NaCl, respectively (Figure 3). At the same levels of salt stress, BRRI dhan54 showed 92 and 160% increase in Pro content compared to control. Importantly, exogenous Pro and GB could also increase the endogenous Pro content further compared to the seedlings exposed to salt without Pro and GB supplementation. However, the comparative increase was higher in salt sensitive BRRI dhan49 than salt tolerant BRRI dhan54 (Figure 3).

**Figure 4:**
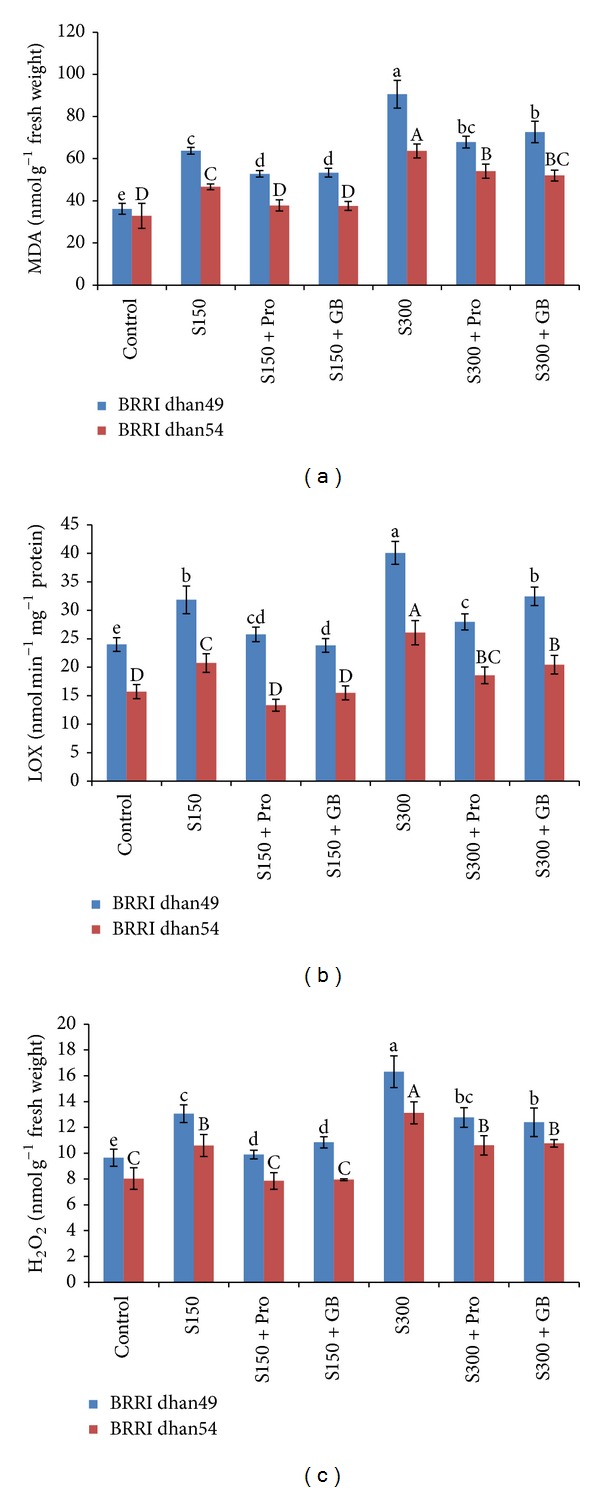
(a) MDA content, (b) LOX activity, and (c) H_2_O_2_ content in salt sensitive and salt tolerant rice seedlings induced by exogenous proline and glycine betaine under salt stress. S_150_ and S_300_ indicate 150 mM NaCl and 300 mM NaCl, respectively. Pro and GB indicate 5 mM proline and glycine betaine spray, respectively. Mean (±SD) was calculated from three replicates for each treatment. Bars with different letters (small letters for BRRI dhan49 and capital letters for BRRI dhan54) are significantly different at *P* ≤ 0.05 applying DMRT.

The MDA content (indicator of lipid peroxidation) sharply increased at any level of salt stress in both rice varieties. However, the rate on increment was higher in salt sensitive BRRI dhan49. In BRRI dhan49, 150 and 300 mM NaCl caused 76 and 159% increase in MDA content while in BRRI dhan54 it was 41 and 95%, respectively, compared to control (Figure 4(a)). The seedlings supplemented with Pro and GB could maintain the level of MDA significantly lower compared to the seedlings exposed to salt stress without supplementation (Figure 4(a)).

In our experiment LOX activity was sharply increased by salt stress (Figure 4(b)). In salt sensitive BRRI dhan49 the LOX activity was increased by 33 and 67% at 150 and 300 mM NaCl, respectively, compared to control, while in salt tolerant BRRI dhan54 the activity was 31 and 65% higher than the control. However, in both of the varieties the activity of LOX was significantly declined in the salt treated seedlings which were supplemented with exogenous Pro and GB (Figure 4(b)).

The levels of H_2_O_2_ also increased noticeably upon exposure to NaCl. In BRRI dhan49 the H_2_O_2_ content was increased by 35 and 69% at 150 and 300 mM NaCl, while in BRRI dhan54 it was increased by 32 and 63%, respectively, compared to control (Figure 4(c)). Both Pro and GB could maintain the H_2_O_2_ content lower in salt-stressed seedlings compared to the seedlings grown without Pro or GB supplementation (Figure 4(c)).

**Figure 5:**
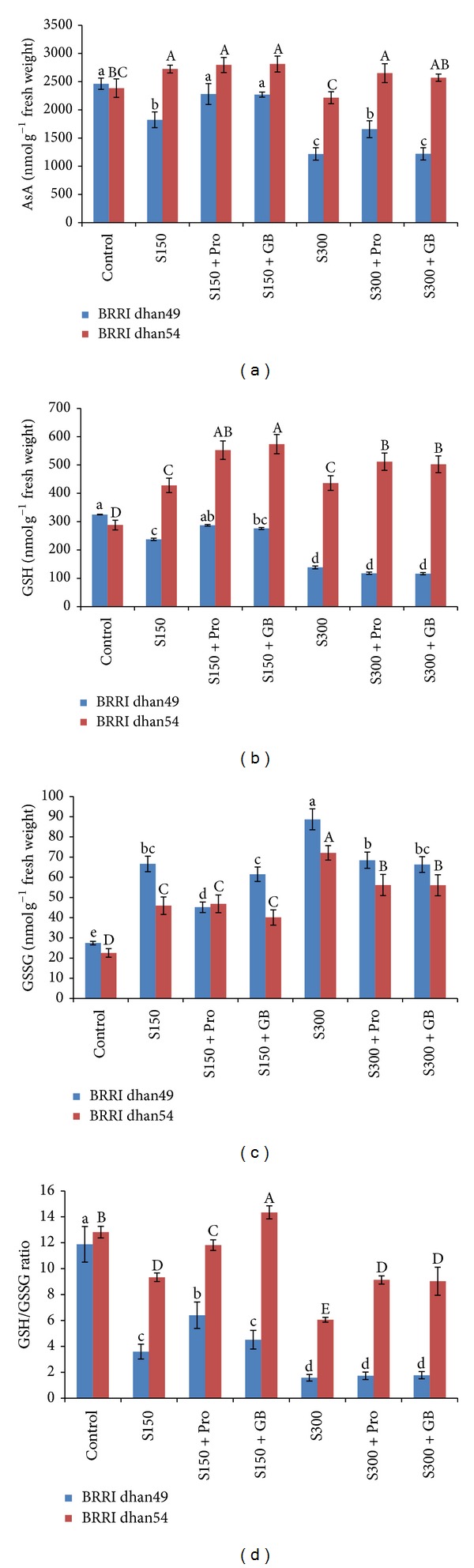
(a) AsA content, (b) GSH content, (c) GSSG content, and (d) GSH/GSSG ratio in salt sensitive and salt tolerant rice seedlings induced by exogenous proline and glycine betaine under salt stress. S_150_ and S_300_ indicate 150 mM NaCl and 300 mM NaCl, respectively. Pro and GB indicate 5 mM proline and glycine betaine spray, respectively. Mean (±SD) was calculated from three replicates for each treatment. Bars with different letters (small letters for BRRI dhan49 and capital letters for BRRI dhan54) are significantly different at *P* ≤ 0.05 applying DMRT.

Leaf AsA content showed differential responses in two rice varieties when exposed to salt stress. In salt sensitive BRRI dhan49 salt stress at any level caused marked decrease in AsA content which was 26 and 51% lower than the control (Figure 5(a)). However, in salt tolerant BRRI dhan54 AsA content increased by 14% at 150 mM NaCl stress, while at 300 mM NaCl it remained unchanged. The seedlings supplemented with exogenous Pro and GB increased the AsA contents in the seedlings of BRRI dhan49 when exposed to salt stress at any level. However, in BRRI dhan54 such protection was observed at 300 mM NaCl only (Figure 5(a)).

In salt sensitive BRRI dhan49 GSH content decreased by 27 and 57% in the seedlings exposed to 150 and 300 mM NaCl, respectively. On the contrary, BRRI dhan54 showed marked increase in GSH content under salt stress which was 49 and 51% higher at 150 and 300 mM NaCl, respectively, compared to control (Figure 5(b)). In BRRI dhan49, the seedlings supplemented with Pro and GB could increase GSH content under mild stress (150 mM NaCl) while no increase was observed in the seedlings grown under 300 mM NaCl. However, in BRRI dhan54 such protection (increase in GSH content) was observed at any level of salt stress (Figure 5(b)).

The GSSG content in rice seedlings of any variety sharply increased at any level of salt stress. In salt sensitive BRRI dhan49 the levels were increased by 143 and 224% at 150 and 300 mM NaCl, respectively. The increase of GSSG was a bit lower in salt tolerant BRRI dhan54 which was 104 and 220% higher at 150 and 300 mM NaCl compared to control (Figure 5(c)). Exogenous Pro and GB, on the other hand, maintained the GSSG content significantly lower under salt stress compared to the seedlings grown without Pro and GB supplementation (Figure 5(c)). However, in tolerant variety BRRI dhan54 Pro and GB did not show this protection under mild salt stress (150 mM).

The ratio of GSH/GSSG decreased markedly under salt stress in dose dependent manners and it greatly varied with varieties (Figure 5(d)). In salt sensitive BRRI dhan49 150 and 300 mM NaCl resulted in 70 and 87% decrease in GSH/GSSG ration, while in salt tolerant BRRI dhan54 it decreased by 21 and 53%, respectively, compared to control (Figure 5(d)).

**Figure 6:**
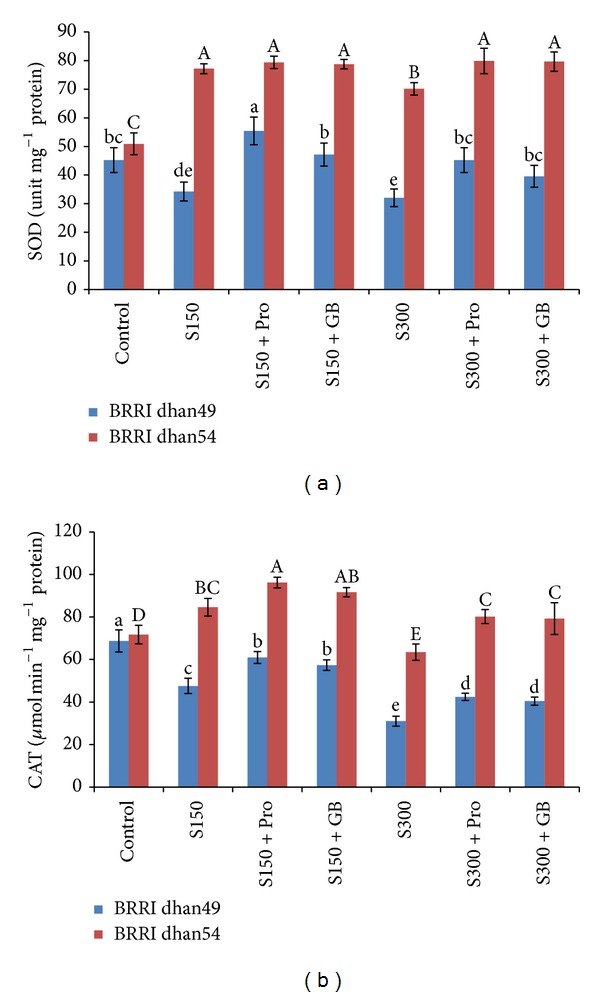
Activities of SOD (a) and CAT (b) in salt sensitive and salt tolerant rice seedlings induced by exogenous proline and glycine betaine under salt stress. S_150_ and S_300_ indicate 150 mM NaCl and 300 mM NaCl, respectively. Pro and GB indicate 5 mM proline and glycine betaine spray, respectively. Mean (±SD) was calculated from three replicates for each treatment. Bars with different letters (small letters for BRRI dhan49 and capital letters for BRRI dhan54) are significantly different at *P* ≤ 0.05 applying DMRT.

Under salt stress the activity of SOD decreased in salt sensitive variety while it increased in salt tolerant variety (Figure 6(a)). In BRRI dhan49 the activity of SOD was 24 and 29% lower than control when treated with 150 and 300 mM NaCl, respectively. On the other hand, the activity was 52 and 38% higher in BRRI dhan54 at same doses of NaCl. In BRRI dhan49, Pro and GB supplemented salt treated seedlings showed the enhancement of SOD activity. However, in case of BRRI dhan54 slight increase in SOD activity was observed only at 300 mM NaCl (Figure 6(a)).

Catalase activity showed differential responses in rice seedlings with variable salt tolerance levels and also induced by salt levels (Figure 6(b)). In salt sensitive BRRI dhan49, the activity decreased by any level of salt stress (31 and 55% lower at 150 and 300 mM NaCl, resp., compared to the control). Salt tolerant BRRI dhan54 showed significant increase in CAT activity under mild stress (150 mM NaCl), whereas a slight decrease (11%) was observed at severe stress (300 mM). However, exogenous Pro and GB enhanced the CAT activity in salt-treated seedlings (Figure 6(b)).

**Figure 7:**
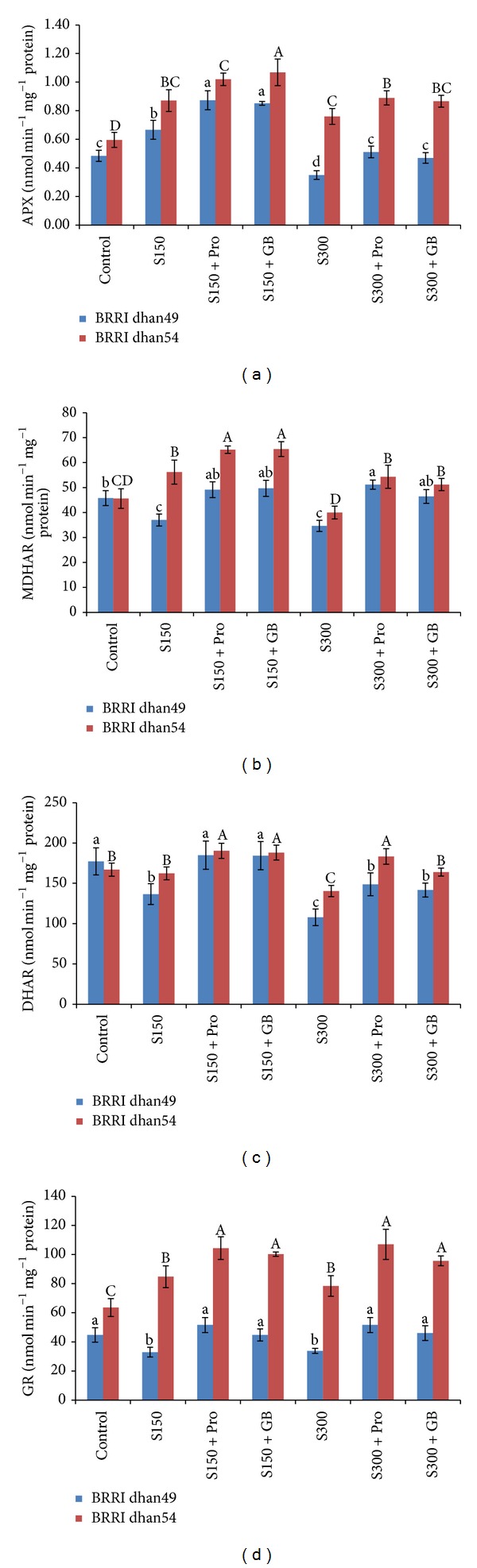
Activities of APX (a), MDHAR (b), DHAR (c), and GR (d) in salt sensitive and salt tolerant rice seedlings induced by exogenous proline and glycine betaine under salt stress. S_150_ and S_300_ indicate 150 mM NaCl and 300 mM NaCl, respectively. Pro and GB indicate 5 mM proline and glycine betaine spray, respectively. Mean (±SD) was calculated from three replicates for each treatment. Bars with different letters (small letters for BRRI dhan49 and capital letters for BRRI dhan54) are significantly different at *P* ≤ 0.05 applying DMRT.

Imposition of salt stress of 150 mM significantly increased the APX activity by 40% in salt sensitive BRRI dhan49 while in salt tolerant BRRI dhan54 it was increased by 45% compared to control. Under severe salt stress (300 mM NaCl), APX activity was decreased by 27% in salt sensitive cultivar but it was increased by 27% in salt tolerant cultivar (Figure 7(a)). Exogenous Pro and GB supplementation in salt stressed seedlings maintained higher APX activities, compared to salt stress alone, whereas in salt tolerant BRRI dhan54 the activity was always higher than BRRI dhan49 (Figure 7(a)). Addition of Pro with 150 mM salt stress increased APX activities in BRRI dhan49 by 29% and in BRRI dhan54 it was as high as the stress condition; its activities were increased by 46 and 17% under severe salt stress in those two cultivars (compared to salt stress alone). The GB addition also increased its activity in all the cases except for BRRI dhan54 in severe stress (Figure 7(a)).

Salt stress at any level decreased the MDHAR activity in salt sensitive BRRI dhan49 which were 19 and 24% lower at 150 and 300 mM NaCl, respectively, compared to control (Figure 7(b)). In contrast, MDHAR activity in BRRI dhan54 increased by 23% at 150 mM NaCl, while it was unaffected under severe salt stress (300 mM NaCl). Exogenous Pro and GB addition under any levels of salt stress significantly increased MDHAR activities irrespective of cultivars (Figure 7(b)).

Salt stress caused a marked decrease in DHAR activity of BRRI dhan49 seedlings at any level of stress; however, in salt tolerant BRRI dhan54 its activity only decreased in severe stress (300 mM NaCl). In BRRI dhan49, due to exogenous Pro application DHAR activities were increased by 35 and 38% at 150 and 300 mM NaCl, respectively. In BRRI dhan54, exogenous Pro supplemented seedlings showed increased DHAR activities by 17 and 31% at 150 and 300 mM NaCl, respectively, compared to salt stress alone (Figure 7(c)). Similarly, in GB supplemented BRRI dhan49 seedlings, DHAR activities were increased by 35 and 31% and its activities increased by 16 and 17% in BRRI dhan54 at 150 and 300 mM NaCl, respectively, compared to salt stress alone (Figure 7(c)).

The GR activity showed different responses in two rice varieties in salt stress. Compared to control, the salt sensitive BRRI dhan49 had decreased GR activities of 26 and 25% in exposure to 150 and 300 mM NaCl, respectively (Figure 7(d)). In opposition, salt tolerant BRRI dhan54 had significantly higher GR activities of 33 and 23% with 150 and 300 mM NaCl, respectively. Nonetheless, exogenous Pro and GB enhanced its activity further in both sensitive and tolerant varieties irrespective of salt doses, compared to the activity in the seedlings exposed to salt stress alone (Figure 7(d)).

**Figure 8:**
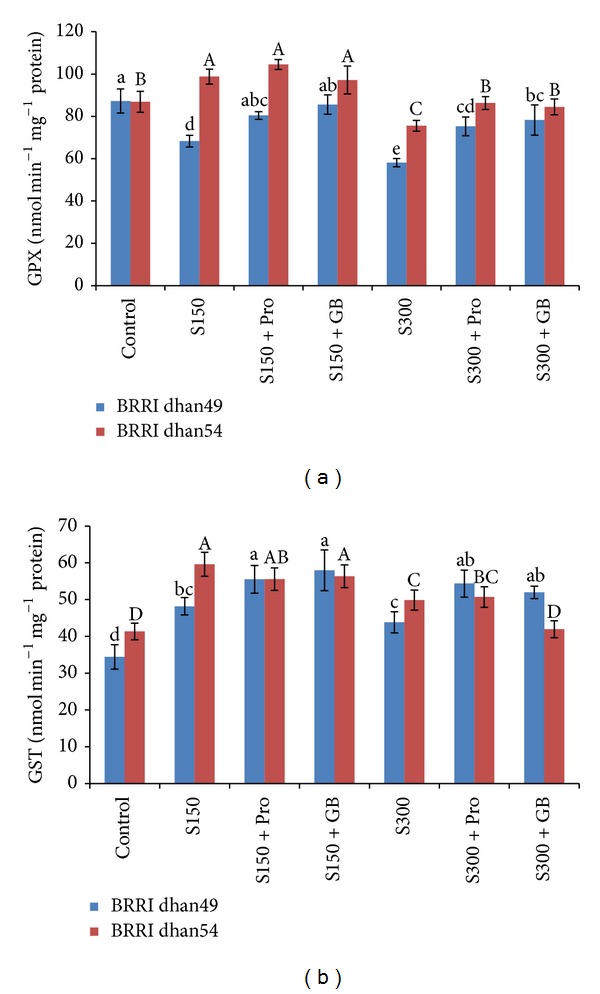
Activities of GPX (a) and GST (b) in salt sensitive and salt tolerant rice seedlings induced by exogenous proline and glycine betaine under salt stress. S_150_ and S_300_ indicate 150 mM NaCl and 300 mM NaCl, respectively. Pro and GB indicate 5 mM proline and glycine betaine spray, respectively. Mean (±SD) was calculated from three replicates for each treatment. Bars with different letters (small letters for BRRI dhan49 and capital letters for BRRI dhan54) are significantly different at *P* ≤ 0.05 applying DMRT.

Salt stresses of any level decreased GPX activity in both salt sensitive and tolerant cultivar, except that mid stress increased GPX activity in BRRI dhan54, compared to control treatment (Figure 8(a)). Compared to 150 and 300 mM salt stress alone, exogenous Pro and GB supplementation increased GPX activity of BRRI dhan49 by 18 and 25%, respectively. In BRRI dhan54 the GPX activity was unaffected by either Pro or GB application (Figure 8(a)). But GPX activity of both salt sensitive and tolerant cultivars was improved significantly by Pro or GB application under severe salt stress (300 mM).

The activity of GST sharply increased in all rice seedlings induced by all levels of salt stress although its activity was slightly higher in salt tolerant BRRI dhan54 (Figure 8(b)). In BRRI dhan49, Pro addition with 150 and 300 mM NaCl resulted in 15 and 24% increases in GST activities, compared to salt stresses alone, while in BRRI dhan54 its activity did not increased further by exogenous application of Pro. Under 150 and 300 mM NaCl, GB increased GST activity of BRRI dhan49 by 20 and 19%, respectively. In BRRI dhan54, exogenous GB addition did not increase GST activity under 150 mM NaCl treatment, while it decreased under 300 mM NaCl (Figure 8(b)).

**Figure 9:**
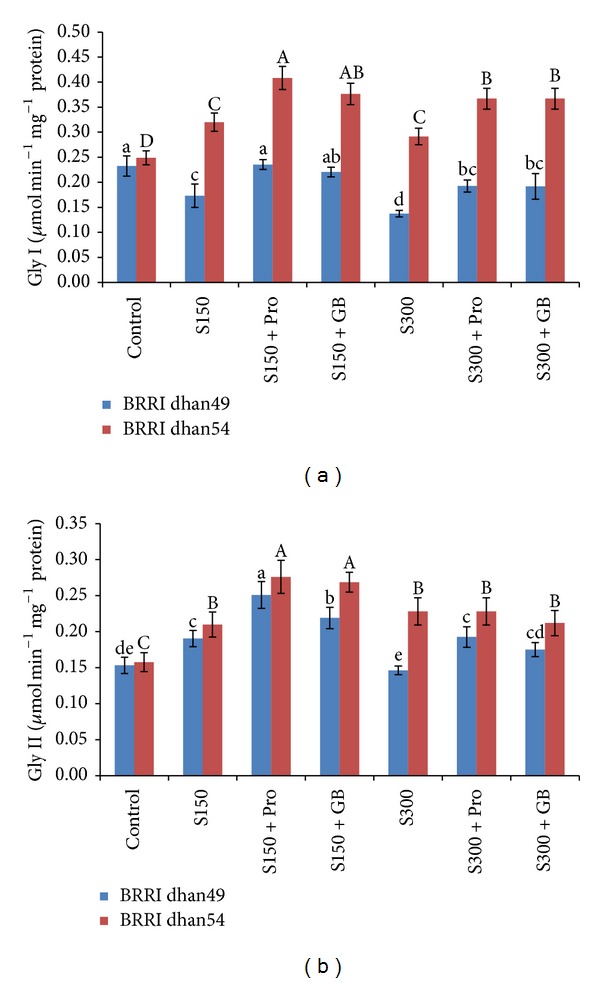
Activities of Gly I (a) and Gly II (b) in salt sensitive and salt tolerant rice seedlings induced by exogenous proline and glycine betaine under salt stress. S_150_ and S_300_ indicate 150 mM NaCl and 300 mM NaCl, respectively. Pro and GB indicate 5 mM proline and glycine betaine spray, respectively. Mean (±SD) was calculated from three replicates for each treatment. Bars with different letters (small letters for BRRI dhan49 and capital letters for BRRI dhan54) are significantly different at *P* ≤ 0.05 applying DMRT.

The activity of Gly I was different in rice varieties differing in salt tolerance. In salt sensitive BRRI dhan49, the activity of Gly decreased by 25 and 41% upon exposure to 150 and 300 mM NaCl, respectively, compared to control (Figure 9(a)). On the contrary, salt tolerant BRRI dhan54 showed 29 and 17% increase in Gly I activity when treated with 150 and 300 mM NaCl, respectively. However, in both of the varieties exogenous Pro and GB enhanced the activity of Gly I further compared to the activity in the seedlings exposed to salt with Pro and GB supplementation (Figure 9(b)).

For salt sensitive BRRI dhan49 the activity of Gly II slightly increased (by 24%) at 150 mM NaCl, while it remained unaffected at 300 mM NaCl. However, in salt tolerant BRRI dhan54 treatment with 150 and 300 mM NaCl resulted in 33 and 45% increase in Gly II activity as compared to control (Figure 9(b)). For BRRI dhan49 Pro and GB supplementation could enhance the Gly II activity at any level of salt (33 and 44% at 150 and 300 mM NaCl, resp.), while in BRRI dhan54 further upregulation of Gly II activity was observed in seedlings grown under 150 mM NaCl (Figure 9(b)).

## 4. Discussion

Salt stress causes several biochemical and physiological alterations such as decrease in water content in tissues, decline in photosynthetic pigments, and oxidative stress. Salt stress often caused accelerated generation and reactions of ROS including ^1^O_2_, O_2_^•−^, H_2_O_2_, and OH^•^ leading to oxidative stress [2]. However, components of antioxidant defense systems in plants work in concert with control the cascades of uncontrolled oxidation and protect plant cells from oxidative damage by scavenging ROS [2, 18]. But in some cases such as severe stress condition these defense systems are required to be upregulated more than their normal limit. Compatible solutes like Pro and GB were found to protect the plants from salt-induced damages due to their role of osmoprotection and antioxidant defense as well. Several reports indicated that enhanced accumulations of Pro and GB are positively correlated with higher tolerance to salt stress [27, 28, 49].

Since salt stress causes osmotic stress, the decline in RWC is a common phenomenon in plants growth under salinity and hence RWC is considered as a potent indicator for evaluating plants for tolerance to salt stress. In our study, salt stress led to a significant decrease of RWC in rice leaves irrespective to NaCl concentration and the rice cultivars (Figure 1). Similar decrease in RWC due to salt stress was reported earlier [50, 51]. Decrease in RWC was due to loss of turgor that results in limited water availability for cell extension processes [52]. However, when salt treated seedlings were supplemented with Pro or GB they showed enhanced RWC which was due to the retention in water in their tissue (Figure 1). The enhanced water content in plants due to exogenous application was also observed by other researchers [22, 53, 54]. In BRRI dhan54 the RWC was slightly higher than BRRI dhan49 which was due to its better tolerance.

In our experiment salt caused reduction in chl content, namely, chl *a*, chl *b*, and chl (*a* + *b*), in both rice varieties. However, the reduction was higher in salt sensitive BRRI dhan49 (Figures 2(a)–2(c)). Salt stress often causes alteration in photosynthetic pigment biosynthesis [55]. Similar decrease in chl content was observed by Amirjani [56] in rice. However, exogenous application of Pro and GB in salt treated seedlings could elevate the chl content which might be due to the higher biosynthesis of the pigment. These results are in agreement with Raza et al. [27] and Sakr et al. [30].

Accumulation of Pro is often suggested as a selection criterion for the stress tolerance of most plant species [22, 24]. In our experiment both rice varieties showed enhanced Proaccumulation under any level of salt stress (Figure 3). However, BRRI dhan54 showed comparatively higher Pro content than BRRI dhan49 due to its adaptive features of higher tolerance. Importantly, exogenous application of Pro and GB in stressed plants further enhanced the endogenous Pro content and in every case the effect of Pro was higher than GB (Figure 3). More importantly in salt tolerant BRRI dhan54 the enhancement due to exogenous Pro and GB was slightly lower than BRRI dhan49 which was probably due to the fact that this variety has got the enhanced level of Pro due to its natural tolerance capacity. Pro and GB-induced counteraction of salt stress due to enhanced Pro content was reported earlier in different plants [27, 30].

Lipid peroxidation is a well-known index for determining the extent of oxidative stress because increased MDA content has been found to be highly correlated with oxidative damages induced by various abiotic stresses including salinity [57]. Hydrogen peroxide is a toxic compound which is injurious to the cell and excessive accumulation of H_2_O_2_ is one of the indicators of oxidative stress [2]. In our experiment both MDA and H_2_O_2_ were found to be increased under salt stress which was in agreement with several previous reports [2, 3, 58–60]. On the contrary, salt treated seedlings supplemented with exogenous Pro and GB showed lower MDA and H_2_O_2_ contents ((Figures 4(a) and 4(c)) which was due to their higher antioxidant defense system. Exogenous Pro and GB-induced upregulation of antioxidant defense and concomitant decrease in MDA and H_2_O_2_ content was observed in many plant species including rice [49, 61, 62]. In our experiment LOX activity was sharply increased in salt treated seedlings in both rice varieties (Figure 4(b)). This higher activity of LOX was assumed as reasons for increased lipid peroxidation that caused oxidation of polyunsaturated fatty acids as reported in many plant studies [63–65]. This result was correlated with higher MDA content of those seedlings grown under salt stress. However, exogenous Pro and GB protected the seedlings by decreasing lipid peroxidation which was reflected with the lower activity of LOX [65].

AsA and GSH have vital roles in development of plant stress tolerance to adverse environmental conditions [66, 67]. Increased AsA or GSH content can effectively reduce ROS produced under stress conditions including salt stress and thus prevents oxidative stress [68]. In the present study, we examined the performance of salt tolerance and salt sensitive rice cultivars against different salinity levels and we also examined how they are protected from salt stress by exogenous Pro or GB application. The result was interesting indeed. Because it was observed that under mild salt stress condition AsA level of salt sensitive BRRI dhan49 was reduced whereas the AsA level of BRRI dhan54 was increased (Figure 5(a)). Severe salt stress also reduced the AsA level of salt sensitive cultivar and this stress maintained the AsA level of tolerant cultivar similar to the control. This result is correlated to MDHAR and DHAR activities which regulate the recycling of AsA within the cell. From Figure 7, it is clear that when the MDHAR or DHAR activity was reduced in salt sensitive BRRI dhan49, then its AsA levels were reduced irrespective of different salt doses. The higher MDHAR and DHAR activities of salt tolerant BRRI dhan54 were also related to its AsA levels (Figures 5(a), 7(b), and 7(c)). Exogenous Pro and GB supplementation significantly enhanced MDHAR and DHAR activities and subsequently improved AsA levels of both cultivars under mild stress. At severe stress, Pro or GB enhanced the activities of two enzymes in both cultivars and tolerant cultivar AsA level was improved in all cases. But GB could not improve the AsA level of sensitive cultivar under severe stress. The similar results regarding modulation of AsA pool and its related enzymes by exogenous Pro and GB under salt stress was previously reported [28, 29]. They also mentioned Pro as more efficient protectant than GB. The GSH/GSSG ratio is indicative of the cellular redox balance that plays vital roles in scavenging ROS, maintains balance state of AsA-GSG cycle, and acts as signal during stress and numerous studies proved its protective roles under stress conditions [10, 18, 69]. In the present study, drastic reduction of GSH contents and increased levels of GSH were observed under salt stress for salt sensitive and salt tolerant cultivars, respectively (Figure 5(c)). Salt stress increased the GSSG levels in both cultivars. These combined rendered the higher GSH/GSSG ratio for tolerant cultivar BRRI dhan54 and reduced GSH/GSSG in sensitive BRRI dhan49 (Figure 5(d)). Exogenous Pro and GB improved GSH and GSH/GSSG ratio in tolerant cultivar in all salinity levels. But in sensitive cultivar these parameters were only improved by Pro under mild salt stress. More efficiency of Pro as compared to GB under salt stress was previously reported [28, 29]. This increased GSH content might be due to the significant increase in GR activities (Figure 7(d)) and higher GSH biosynthesis [18]. Previously higher reduced glutathione GSH content and GSH/GSSG ratio proved the existence of reduced redox state in cells by exogenous Pro and GB treatment [28, 70]. Previous research findings also expressed that higher levels of GSH or GSH/GSSG ratio induced by exogenous Pro and GB in turn helps to maintain the activity of GSH dependent antioxidant enzymes such as GPX, GR, and GST to scavenge ROS from cell [28, 70, 71]. These findings supported the results of our study which are mostly factual for the salt tolerant BRRI dhan54 and in some cases for the sensitive BRRI dhan49.

Salt stress-induced excess generation of ROS and subsequent enhanced activities of many antioxidant enzymes during salt stress have been reported in many plant species. Importantly, the activities of antioxidant enzymes of salt tolerant genotypes are upregulated under salt stress where salt sensitive species failed to do so [2, 4, 72]. In our experiment antioxidant enzymes responded differently in two rice varieties grown under two levels of salt stress. Exogenous application of Pro and GB also showed their protective effect differently in those varieties. Superoxide dismutase is the first line of antioxidant enzyme that removes O_2_^•−^ by catalyzing its dismutation, one O_2_^•−^ being reduced to H_2_O_2_ and another oxidized to O_2_ [4]. The enhanced activity of SODs minimizes abiotic oxidative stress and has a significant role in the adaptation of a plant to stressed environments [4]. In our experiment salt stress downregulated the SOD activity in salt sensitive BRRI dhan49 while it clearly upregulated in salt tolerant BRRI dhan54. However, Pro and GB supplementation could enhance the activity further which indicated its role in ROS detoxification (Figure 6(a)). Catalase is a potential enzyme which has higher turnover rate and is capable to dismutase two molecules of H_2_O_2_ to water and oxygen and thus is considered as an efficient ROS detoxifier [4]. There are plenty of reports on the changes in CAT activity or expression and those supported the notion that CAT is the most efficient H_2_O_2_ scavenging enzyme [4, 73]. In our experiment, salt sensitive BRRI dhan49 showed decline in CAT activity at any level of NaCl which might be due to ineffective enzyme synthesis or change in assembly of enzyme subunits (Figure 6(b)) [74, 75]. On the contrary, in salt tolerant BRRI dhan54 the activity slightly increased under mild salt stress (150 mM NaCl) but decreased under severe stress (300 mM NaCl). This trend was supported by earlier reports [58, 76, 77]. However, the activity of CAT in the presence of Pro and GB under salt treatment was much higher than those under salt treatment without Pro and GB which suggest a unambiguous role of Pro and GB in scavenging H_2_O_2_ under salt stress. Similar effects were also observed in several recent studies [28, 31, 49, 78].

The four enzymes of AsA-GSH, namely, APX, MDHAR, DHAR, and GR, are vital for antioxidant defense because they are involved in maintaining the AsA and GSH pool. In our experiment APX activity increased in both rice varieties subjected to salt stress and exogenous Pro and GB enhanced the activity further which indicated the H_2_O_2_ scavenging role of Pro and GB (Figure 7(a)). This result was in agreement with other findings [6, 25, 49]. MDHAR and DHAR are two important enzymes related to the regeneration of AsA which is a strong antioxidant. Both enzymes are equally important in regulating AsA level and its redox state under oxidative stress condition [79–81]. The activity of MDHAR and DHAR clearly decreased in salt sensitive BRRI dhan49 treated with NaCl. However, in salt tolerant BRRI dhan54 the activity increased or remained unchanged depending on the salt concentration (Figures 7(b) and 7(c)). Decrease of MDHAR and DHAR activity under salt stress was also reported in our earlier studies [2, 3]. Exogenous Pro and GB supplemented seedlings, on the other hand, enhanced the activity in both varieties which helped the plants in efficient regeneration of AsA (Figure 5(a)). Glutathione reductase is another important enzyme of AsA-GSH cycle which is important for maintaining high ratio of GSH/GSSG in plant cells, also necessary for accelerating the H_2_O_2_ scavenging [82, 83]. In salt sensitive BRRI dhan49 the activity of GR declined under salt stress in dose dependent manners but in salt tolerant BRRI dhan54 it declined only at 300 mM NaCl (Figure 7(d)). Higher activity of GR in stress tolerant plants was observed in several studies [76, 84]. However, in both rice varieties, supplementation of Pro and GB in salt treated seedlings showed enhanced activities of GR which could maintain a high GSH pool (Figures 5(b) and 7(d)). Our results were corroborated with other recent findings where exogenous Pro and GB upregulated the GR activity under salt stress [15, 25, 28].

The GPX is another vital enzyme of antioxidant defense system and due to substrate specifications and stronger affinity for H_2_O_2_ it can efficiently scavenge, especially, H_2_O_2_ and thus provide protection against stress [4, 85]. In our experiment salt tolerant variety showed higher activities of GPX compared to salt sensitive variety which was due to an increased synthesis of the enzymes or an increased activation of constitutive enzyme pools (Figure 8(a)). In salt sensitive variety GPX activity declined at any level of salt stress. This indicates inefficient detoxification of ROS in salt sensitive variety (BRRI dhan49). Differential response of GPX activity in salt sensitive and tolerant varieties was reported in many plant studies [2, 3, 86]. However, in salt sensitive variety exogenous Pro and GB upregulated the activity of GR at any level of stress. In contrary, in salt tolerant variety (BRRI dhan54) Pro and GB supplemented seedlings showed further increase in GR activity only at severe stress which indicates that under mild stress the activity was well enough to scavenge the excess H_2_O_2_ (Figures 8(a) and 4(c)). It is interesting that GST activity increased sharply in both of the rice varieties exposed to NaCl. Although the primary role of this enzyme has been assigned to the detoxification of xenobiotics, it has also been shown to exhibit antioxidant activity [4]. Plant GSTs are also associated with responses to various forms of abiotic stress [45, 87] and stress tolerance is often correlated with enhanced activity of GST [4]. In both rice varieties of our experiment, GST activity markedly increased under salt stress where comparatively higher activity was observed in salt tolerant BRRI dhan54 (Figure 8(b)). Interestingly, exogenous Pro and GB only resulted in further enhancement of GST activity in BRRI dhan49 only which indicates that the salt tolerant BRRI dhan54 has already got enough capacity to detoxify H_2_O_2_ (Figures 8(b) and 4(c)). Our results are partially supported by Hoque et al. [29].

Glyoxalase system consisting of Gly I and Gly II enzymes effectively detoxify MG in two-step reactions where GSH acts as a cofactor. In first step, Gly I catalyzes the formation of S-d-lactoylglutathione (SLG) from the hemithioacetal formed nonenzymatically from MG and GSH. In second step, Gly II catalyzes the hydrolysis of SLG to regenerate GSH and release d-lactate [17, 88]. Effective glyoxalase system converts MG to d-lactate and at the same time it recycles GSH properly [2, 3]. Several studies proved that efficient glyoxalase system not only detoxifies MG, but also improves stress tolerance [10, 18]. Different levels of salt stresses significantly reduced Gly I activities in salt sensitive BRRI dhan49. Decrease in Gly I activity under salt stress was also reported previously [2, 89]. On the contrary those stresses enhancing Gly I activity in salt tolerant BRRI dhan54 cultivar might be an indication of its better tolerance capacity. Moreover, exogenous Pro and GB supplementation with salt media improving Gly I activity in two cultivars in different stress conditions proves the enhanced tolerance level induced by Pro and GB (Figure 9(a)). After salt exposure Gly II activity of salt sensitive BRRI dhan49 was increased only under mild salt stress. On the other hand tolerant BRRI dhan54 increased its activity in both stress levels (Figure 9(b)). Enhanced Gly II activity in response to salt stress was also observed in our previous study [90]. When these cultivars were supplied with exogenous Pro or GB with salt stress their Gly II activities significantly enhanced. Pro- and GB-induced upregulation of glyoxalase enzymes and subsequent salt stress tolerance were also reported by other researchers [29, 32]. Engineering of glyoxalase pathway enzymes in different plant species reduced MG content under salt that was correlated to GSH-based MG detoxification system which reduced oxidative damage [16, 17, 91–93]. These results are in line with our findings. If the glyoxalase enzymes of two cultivars are compared it can be said that the tolerant BRRI dhan54 had higher Gly I and Gly II activities in all the cases, compared to BRRI dhan49. Even if the GSH level of these two cultivars are compared it is clear that BRRI dhan54 is better performer as it maintained higher GSH levels both under salt stress conditions with or without Pro and GB (Figure 5(b)). The higher GSH level with higher Gly I and Gly II activities are evidence for efficient glyoxalase system for conferring salt stress tolerance [18, 90]. Thus higher Gly I and Gly II activities with higher GSH level induced by Pro and GB have made BRRI dhan54 more salt tolerant cultivar than BRRI dhan49.

## 5. Conclusion

Considering the above results we, therefore, conclude that exogenous Pro and GB are effective protectants to improve short-term salt tolerance both in salt sensitive BRRI dhan49 and salt tolerant BRRI dhan54 as Pro and GB effectively maintained better physiological conditions and significantly alleviated oxidative damages of rice seedlings by enhancing the antioxidant and glyoxalase systems. In all the cases BRRI dhan54 was a better performer under salt stress. Although the studied antioxidant and glyoxalase enzymes were improved by both the osmoprotectants; the GB could not restore the nonenzymatic antioxidants of salt sensitive cultivar BRRI dhan49 in some cases. Considering these facts it can be assumed that Pro is good for salt sensitive cultivar at present studied condition. From the comparative studies of salt tolerant and sensitive cultivars, it can be said that enhancement of tolerance by Pro and GB even in the salt sensitive cultivar is an interesting point and this result deserves further intrinsic researches. The dose duration dependent study with Pro and GB in different salinity levels might be elucidated. Mechanisms of Pro and GB as osmoprotectants, free radical scavengers, enzyme activators, or as regulators of other physiological processes were stated in many articles but merely studied under different stress conditions. Studies on their protective mechanisms and signaling cascades are the further scope of research.
